# Stigma and Help-Seeking Attitudes in Relation to Psychological Distress Among Medical Students in Saudi Arabia

**DOI:** 10.2174/0117450179414477250905100917

**Published:** 2025-09-11

**Authors:** Wejdan M. Al-Johani, Abdulelah H. Almansour, Dalal M. AlBakr, Duaa Alghirash, Abdulmohsen N. Alfadhli, Raseel B. Almutairi, Osama A. Mobarki, Sultan A. Alqasim, Malak A. Al-Shammari, Moataza M. Abdelwahab

**Affiliations:** 1 Department of Family and Community Medicine, College of Medicine, Imam Abdulrahman Bin Faisal University, Dammam, Saudi Arabia; 2 Department of Psychiatry, College of Medicine, Imam Abdulrahman Bin Faisal University, Dammam, Saudi Arabia; 3 Imam Abdulrahman bin Faisal University, College of Medicine, Dammam, Saudi Arabia; 4 College of Medicine, Jazan University, Jazan, Saudi Arabia; 5 College of Medicine, King Saud bin Abdulaziz University for Health Sciences, Riyadh, Saudi Arabia

**Keywords:** Help-seeking, Mental health, Medical students, Psychological distress, Psychological stigma, Saudi Arabia

## Abstract

**Introduction:**

Psychological distress is common among medical students worldwide. However, psychological stigma remains a significant barrier to seeking help. This study explores the association between psychological distress, stigma, and attitudes toward help-seeking among medical students in the unique cultural context of Saudi Arabia.

**Methods:**

A cross-sectional study was conducted using an online survey distributed to medical students across Saudi Arabia, yielding 1,077 completed responses. Sociodemographic data were collected, along with responses to the Kessler Psychological Distress Scale (K6), the Stigma Scale for Receiving Psychological Help (SSRPH-A), and the Attitudes Toward Seeking Professional Psychological Help Scale (ATSPPH-SF-A). Data were analyzed using descriptive statistics, Spearman’s correlation, and factor analysis.

**Results:**

Overall, 32.2% of students reported low distress, 34.5% moderate distress, and 33.4% high distress. Female students exhibited higher distress but greater openness to seeking help, while males reported higher stigma. Lower GPA and income were associated with increased distress and reduced help-seeking attitudes. Regional variations were observed, with Najran reporting the highest distress, and Madinah and Qassim showing higher openness to help-seeking.

**Discussion:**

Findings are consistent with the international studies, indicating that stigma significantly hinders service utilization despite need. Cultural norms, socioeconomic status, and educational systems further shape attitudes. Digital healthcare transformation in Saudi Arabia offers opportunities to reduce stigma and expand access.

**Conclusion:**

Psychological distress and stigma remain prevalent among Saudi medical students, influenced by gender, socioeconomic, and regional factors. Targeted, culturally informed interventions are essential to reduce psychological stigma, promote awareness, and encourage positive help-seeking behaviors within academic environments.

## 1 INTRODUCTION

Psychological distress refers to a state of emotional discomfort characterized by symptoms such as anxiety, low mood, fatigue, sleep disturbances, and physical complaints like headaches or restlessness [1-3]. Medical students specifically are at high risk of psychological distress due to their exposure to intense academic demands and emotionally challenging environments [4-8]. In Saudi Arabia, as in many countries, medical education is associated with increased rates of anxiety, depression, and stress-related symptoms, which can negatively impact students' academic performance and overall well-being [9-11]. A study conducted at King Saud University in Riyadh found that 56.3% of medical students experienced moderate to severe psychological distress, highlighting the significant mental health challenges faced by this group [12]. These results emphasize the importance of promoting mental well-being within medical education settings in Saudi Arabia.

Mental health stigma is defined as “the disgrace, social disapproval, or social discrediting of individuals with a mental health problem” [13-16]. Cultural factors heavily influence stigma surrounding mental illnesses [17-20]. In Saudi Arabia, common cultural factors include deeply rooted traditions, societal norms, familial upbringing, and a general lack of mental health awareness within the community. Additionally, several studies assumed that the spiritual background of Saudi society has shaped common beliefs attributing mental disorders to supernatural causes, personal weakness, lack of faith, or black magic (*e.g.*, the “evil eye”) [21-25]. Cultural misconceptions also lead to fear of aggressive behavior influenced by psychiatric conditions and concern about the adverse effects of psychiatric medications. Furthermore, mental disorders are often perceived as either hereditary or incurable, which reinforces negative attitudes and reluctance toward seeking psychological help [26,27].

Furthermore, medical students specifically often fear being labeled as weak or unfit for the profession, which leads them to suppress their emotional struggles rather than seeking professional help [28, 29]. Likewise, a study on Egyptian adolescents found that stigma, self-perception, and lack of time were commonly reported obstacles to seeking help, even when severe psychological symptoms were present [7]. In Saudi Arabia, a study reported that 34% of the general population who participated in the survey met the criteria for mental health disorders, but only 5% of them sought help, which signifies substantial reluctance toward seeking treatment [30,31]. Notably, the more distressed individuals felt, the more likely they were to perceive stigma, creating a feedback loop where worsening mental health leads to even greater reluctance to seek support [32].

Despite existing mental health services and outreach efforts, psychological distress remains underdiagnosed and undertreated among Saudi medical students. This disconnection between the available care and actual utilization signals the need for deeper investigation into how mental health stigma influences help-seeking attitudes. Therefore, this study aims to explore the association between psychological distress, perceived stigma, and help-seeking attitudes among medical students in Saudi Arabia. It addresses an existing research gap by assessing the prevalence of distress and investigating how sociodemographic factors may influence these associations. Understanding this relationship is crucial for developing culturally informed, accessible, and effective interventions.

## 2 METHODS

### 2.1 Study Population and Sampling

This cross-sectional study targeted Saudi medical students across the 13 regions of Saudi Arabia. The inclusion criteria were Saudi medical students in any academic year, encompassing both males and females enrolled in governmental or private universities. The exclusion criteria comprised individuals who were not medical students, non-Saudis, or those studying outside Saudi Arabia. Assuming a 50% positive attitude toward seeking psychological help, the minimum required sample size was 1066, calculated using Epiinfo version 7.0. This obtains a margin of error of 3% at a confidence level of 95%. A total of 1077 responses were received.

### 2.2 Data Collection

An online self-administered questionnaire was designed on QuestionPro software and distributed through representatives in each college. The survey was customized to accept a single response from each participant to avoid duplication of responses. The first part included the sociodemographic information (age, gender, educational level, marital status, income, and GPA).

The second part included questions to measure psychological distress by a six-item scale (Kessler psychological distress scale) (K6), which is the most widely used screening scale of non-specific psychological distress, providing an estimated prediction of the prevalence of serious mental illness in the past 12 months. Each item is scaled from 0 to 4, with zero meaning “none of the time” and four meaning “all of the time.” Total scores range from 0 to 24 and are further divided into three categories: scores between 13 and 24 represent high psychological distress, scores between 8 and 12 indicate moderate psychological distress, and scores between 0 and 7 represent low psychological distress.

The third part consisted of Stigma Scales for Receiving Professional Psychological Help (SSRPH-A). It is a 5-item self-reported instrument that measures the stigma associated with receiving psychological help. Items are rated on a 4-point Likert-type scale ranging from 1 (“strongly disagree”) to 4 (“strongly agree”). The SSRPH-A total score is obtained by summing up the five items, with higher scores indicating a higher degree of perceived stigma.

The Fourth part, the Attitudes Toward Seeking Professional Psychological Help Scale-Short Form (ATSPPH-SF-A), is a 10-item scale that evaluates participants' attitudes toward seeking professional help for psychological problems. The items are rated on a 4-point Likert-type scale ranging from 0 (“disagree”) to 3 (“agree”). The total ATSPPH-SF-A score is obtained by reversing the scores of items 2, 4, 8, 9, and 10. Higher scores indicated a more positive attitude.

### 2.3 Data Analysis

The collected data were analyzed using the IBM SPSS software version 26.0. Statistical significance was set at p<0.05. For validation of the above three scales, a Principal Component Analysis (PCA) was used. The PCA resulted in one component for psychological distress and one component for Stigma questions, while the attitude scale loaded into two components, namely, Openness to seeking treatment and Value and need in seeking treatment, all with factor loading of more than 0.5. Subsequently, Cronbach's alpha was used to determine the internal consistency of the items within each factor.

Descriptive statistics were displayed, namely frequency distribution for categorical data, while continuous data were presented using range, mean, standard deviation, and median, as well as boxplots. A correlation analysis was performed between the scores of distress, stigma, and attitude (openness and value), as well as age and GPA, using Spearman's correlation. Factors related to distress, stigma, and attitude components were examined using the Mann-Whitney test for binary variables and the Kruskal-Wallis test for other categorical variables, with all pairwise comparisons adjusted for using the Bonferroni correction.

## 3 RESULTS

A total of 1077 medical students and interns from the main 13 regions of Saudi Arabia participated in the study. The highest responses were from Riyadh, with 337, followed by the Eastern region, with 280 responses, and Jazan, with 251 responses. The age of the participants ranged from 17 to 25 years, with a mean age of 22 (Mean SD =21.6 ± 1.7) years. The gender distribution was approximately equal, with males representing 53.8% (579) of the total sample and females representing 46.2% (498). The majority of participants were single (N = 1043, 96.8%). About one quarter of the total sample reported a family income of <10000 SAR (N=277, 25.7%). Approximately 95% of them were from governmental universities. Out of the total sample, 838 (77.8%) follow the Problem-Based Learning (PBL) teaching method. Only 134 (12.4%) of the total participants admitted failure during medical school (Table **1**).

Table **2** displays the factor loadings for individual questions across the three scales used (Psychological Distress, SSRPH, ATSPPH-SF). Psychological distress and Stigma questions were loaded into one component each, while the attitude scale loaded into two components, the first component is Openness to seeking treatment, and the second component is Value and need in seeking treatment. Cronbach's Alpha coefficients are provided to assess the internal consistency of each scale. The Distress scale demonstrates good reliability (α = 0.854). The Stigma scale has acceptable reliability (α = 0.7), while the Openness scale shows marginal reliability (α = 0.619). The Value-Added scale exhibits acceptable reliability (α = 0.706).

**Fig. (1):**
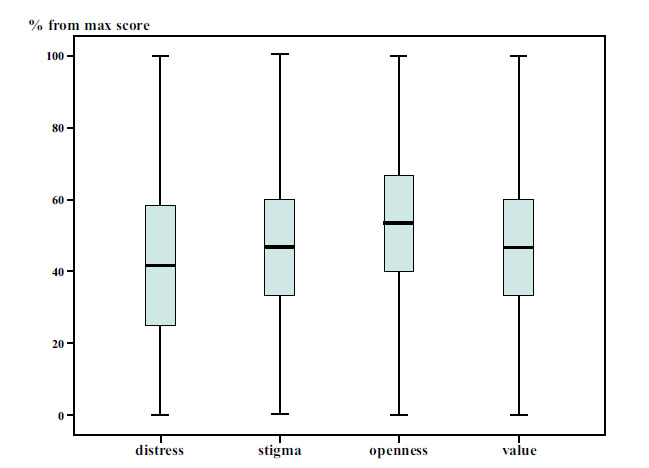
Percentages from the maximum score of distress, stigma, and attitudes (openness and value).

Out of the 1,077 participants, 32.2% (95% CI: 29.5%–35.0%) reported low distress, 34.5% (95% CI: 31.7%–37.4%) reported moderate distress, and 33.4% (95% CI: 30.6%–36.3%) reported high distress. (Fig. **1**) presents boxplots showing the distribution of students' scores for psychological distress, perceived stigma, and attitudes toward seeking psychological help (openness and value), expressed as percentages of the maximum possible scores. Distress scores ranged from 0% to 100% of the maximum (24), with a median score of 10, corresponding to 41.7% of the maximum, which indicates a moderate overall level of psychological distress among participants. Stigma scores ranged from 0% to 100% of the maximum (15), with a median of 7, equivalent to 46.7% reflecting a moderate level of perceived stigma regarding receiving psychological help. The maximum score for openness is 15. The student's score ranged from 0 (0%) to 15 (100%), with a median of 8 (53.3%). While the maximum score for Value is 15, the student's score ranged from 0 (0%) to 15 (100%), with a median of 7 (46.7%).

Table **3** presents the correlation of variables, including age, distress, stigma, and attitude components (openness and values). These findings suggest that older individuals experience less psychological distress and perceive a higher value in seeking help.Additionally, higher stigma is correlated with greater distress, but with lower openness and lower perceived value. Similarly, higher distress is associated with greater openness, but with lower perceived value.

Table **4** demonstrates the association between sociodemographic factors and the scores of distress, stigma, and attitude components (openness and values). The study's results revealed that, except for stigma, all variables were influenced by the participants' place of residence. Notably, Najran scored the highest levels of distress (MD = 14), while Madinah and Qassim have significantly high openness to seeking medical help (MD = 10). Conversely, Tabuk scored the lowest perceived value of seeking medical advice compared to other regions.

The study revealed that females had higher psychological distress and were more open to seeking help, whereas males showed a greater tendency towards stigmatization. Marital status showed no difference among the variables studied.

Regarding the average family income, the participants with a monthly family income <10000 SAR showed a lower help-seeking attitude in terms of openness and perceiving the value, than those with a monthly family income >20000 SAR.

Students in private universities showed higher distress and higher scores of perceived value in seeking help. Additionally, those taught *via* PBL had higher scores in the perceived value of seeking help. Those who failed at least one exam in medical school had higher distress and higher stigma scores. Regarding students' GPA grades, students with lower GPAs were more psychologically distressed (rs=-0.107, p=0.0001), and had higher scores for Value in seeking help (rs=-0.061, p=0.047)

## 4 DISCUSSION

The interplay between psychological distress and stigma with the attitudes towards seeking help remains a critical focus of mental health research. Interestingly, this study indicates that students experience moderate levels of psychological distress and stigma, alongside a moderate willingness to access assistance. Almost two-thirds of the study participants are suffering from moderate to high levels of distress, which is consistent with the findings of a study conducted in 2019 in Riyadh [12]. This reinforces the claim that psychological distress remains a common and growing concern among medical students in Saudi Arabia [12]. A study conducted in the UK reported that 50% of medical students are experiencing moderate to severe somatic symptoms along with depressive and anxiety disorders [33]. Similarly, in Iran, Egypt, Qatar, and Sudan, high levels of distress were reported (61%, 60%, 40%, and 32%, respectively) [34-37]. These results demonstrate that medical students worldwide are vulnerable to considerable psychological distress, showcasing the necessity for multinational strategies to combat the situation.

In this study, participants reported a moderate level of stigma, which aligns with both local and international research [38-42]. A previous study found that stigma is considered one of the significant barriers to the actual utilization of mental health services among medical students despite a high perceived need [43]. Consistently, findings on Chinese college students revealed that the increased mental health literacy enhances the seeking of professional psychological help [44]. This emphasizes that stigma prevents even distressed medical students from seeking professional help, suggesting that overcoming stigma and improved support systems could foster more positive attitudes toward help-seeking, ultimately enhancing their mental well-being and health outcomes [39, 45].

The older participants tend to experience lower levels of psychological distress and a higher perceived value for seeking help. This suggests that as individuals age, they are more likely to recognize the importance of seeking professional psychological help and experience less psychological distress. Furthermore, higher levels of stigma are associated with greater distress, reduced openness to seeking help, and a diminished perceived value of professional psychological help. This aligns with the findings of a study among Saudi adults, in which stigma negatively influences help-seeking attitudes [38]. In this regard, multiple studies worldwide have reported that stigma can significantly influence an individual's willingness to seek treatment, often leading to delayed presentation, treatment avoidance, or non-adherence to the treatment plan [46-51].

Regarding the variations among different regions of Saudi Arabia, the analysis revealed that psychological distress levels were highest among students in Najran, while openness to seeking psychological help was notably higher in Madinah and Qassim. Conversely, students in Tabuk exhibited the lowest perceived value in seeking help. These regional differences may reflect varying cultural attitudes, availability of mental health resources, and institutional support systems [52]. For instance, disparities in mental health service utilization across regions have been documented nationally and internationally, highlighting the need for region-specific interventions [52, 53]. However, Saudi Arabia is undergoing a rapid digital transformation of its healthcare system, which presents valuable opportunities to improve mental healthcare [54-56]. The privacy and anonymity offered by digital platforms can reduce stigma and encourage a help-seeking attitude [54].

Consistent with prior research, female students reported higher levels of psychological distress and were more likely to acknowledge and seek help for their psychological problems [52]. In contrast, male students demonstrated a higher tendency toward stigmatization, which may contribute to the underutilization of mental health services among this group [57]. This outcome may be attributed to the cultural framework in Saudi Arabia, where seeking psychological help is often perceived as a sign of male weakness or a potential source of shame for both the individual and his family. Therefore, cultural expectations surrounding masculinity and traditional male roles may discourage men from pursuing mental health support, as doing so may conflict with socially accepted norms of male behavior [58, 59]. Moreover, several studies have compared the stigma level and its impact among people from different cultural backgrounds [60]. For instance, there are higher levels of stigma in Canada and Korea than in Saudi Arabia, which led to more delayed presentation among Canadian participants [61]. This was explained by the involvement of Asian families in patient care, which could potentially reduce the level of stigma from the patients' perspective [17, 61]. Additionally, cultural impact has also been reported within African countries. For instance, studies have found the highest prevalence of stigma in Ethiopia, followed by Egypt and Nigeria, which indicates that cultural factor plays a crucial role in mental health stigmatization worldwide [62-66].

Regarding socioeconomic status, this study revealed that lower family income was correlated with a negative help-seeking attitude, characterized by being less open to seeking help and perceiving less value in doing so. This suggests that financial constraints and associated stressors may hinder help-seeking attitudes as reported by prior studies conducted worldwide [67-69]. Previous studies have shown that lower socioeconomic status is linked to increased psychological distress and reduced access to mental health resources. This may also be a contributing factor to the discrepancy in mental health service utilization among different geographical areas in the country [58, 70].

Students attending private universities reported higher distress levels and greater perceived value in seeking help, possibly due to increased academic pressures and financial burdens associated with private education. Additionally, students engaged in Problem-Based Learning (PBL) curricula exhibited higher scores in the perceived value of seeking help. This can be attributed to the collaborative and reflective nature of PBL, which fosters greater awareness of mental health needs. This has been cited in a few studies in the region and abroad, emphasizing that the modality of teaching during academic training may play a crucial role in normalizing the utilization of mental health services during these years and later [71, 72]. Furthermore, similar to previous studies, this study found a negative correlation between GPA and psychological distress, indicating that lower academic achievement is associated with higher mental health challenges [73]. These findings are also aligned with the literature highlighting the impact of academic stress on medical students' mental well-being [74-76].

## 5 STUDY LIMITATIONS

This study provides valuable insights. However, several limitations should be acknowledged. The cross-sectional design restricts the ability to establish temporal or causal relationships. Additionally, the reliance on self-administered questionnaires may introduce biases, including recall bias and social desirability bias, where participants might respond in a manner they perceive as culturally favorable. The restricted sample size limits the ability to generalize the findings to the overall medical student population. Moreover, important confounding variables such as academic pressure, family history of mental illness, and access to mental health services were not assessed due to the predefined scope of the study and limitations of the survey instrument. Future research should consider including these factors to provide a more comprehensive understanding of psychological distress and help-seeking behavior. Moreover, longitudinal studies on larger populations are also recommended to explore causal relationships between sociodemographic factors and mental health outcomes among medical students.

## CONCLUSION

This study reported high levels of psychological distress and stigmatization among medical students, which correlate negatively with help-seeking attitude. The study's findings emphasize the need for targeted mental health interventions that consider regional, gender, and socioeconomic differences among medical students in Saudi Arabia. Universities should implement culturally sensitive programs to reduce stigma and promote help-seeking, particularly among male students and those from lower-income backgrounds. Additionally, integrating mental health education into medical curricula and providing accessible support services can help address the identified disparities.

## Figures and Tables

**Table 1:** Sociodemographic and academic characteristics of Saudi medical students 2025.

		No (n=1077)	%
Residence	Riyadh	337	31.30%
	Eastern region	280	26.00%
	Jazan	251	23.30%
	Makkah	58	5.40%
	Aseer	32	3.00%
	Tabuk	28	2.60%
	Madinah	25	2.30%
	Qasseem	16	1.50%
	Aljouf	14	1.30%
	Baha	13	1.20%
	Northern Border	10	0.90%
	Hail	8	0.70%
	Najran	5	0.50%
Age (years)	Min-max Mean SD median	17-25 21.6 ± 1.7 22	
Gender	Male	579	53.8%
	Female	498	46.2%
Marital Status:	Single	1043	96.8%
	Married	28	2.6%
	Divorced	4	0.4%
	Widowed	2	0.2%
Family monthly income	<10000 SAR	277	25.7%
	10000-20000 SAR	323	30.0%
	> 20000 SAR	477	44.3%
Type of university	Private	55	5.1%
	Governmental	1022	94.9%
Method of teaching	PBL	838	77.8%
	traditional curriculum	239	22.2%
Current year of study	2nd year	205	19.0%
	3rd year	185	17.2%
	4th year	154	14.3%
	5th year	242	22.5%
	6th year	245	22.7%
	Internship	46	4.3%
Last known GPA?	A+	292	27.1%
	A	266	24.7%
	B+	218	20.2%
	B	138	12.8%
	C+	77	7.1%
	C	47	4.4%
	D+	21	1.9%
	D	10	0.9%
	F	8	0.7%
Ever failed during med school	Yes	134	12.4%
	No	943	87.6%

**Table 2:** Factor loading and reliability of (psychological distress, SSRPH, ATSPPH-SF) scales.

Scale	Psychological Distress		Stigma		Attitude (ATSPPH-SF)		
Component	-	-	-	-	-	openness	value
-	Q4	.814	Q3	.786	Q3	.728	.105
-	Q2	.814	Q5	.750	Q5	.715	.160
-	Q6	.761	Q4	.655	Q6	.698	.082
-	Q5	.752	Q1	.638	Q1	.630	.055
-	Q3	.737	Q2	.541	Q7	.573	-.060
-	Q1	.686	-	-	Q9	.150	.717
-	-	-	-	-	Q10	.113	.648
-	-	-	-	-	Q8	-.008	.632
-	-	-	-	-	Q2	.285	.576
-	-	-	-	-	Q4	-.115	.528
Cronbach's Alpha	0.854		0.7		-	0.619	0.706

**Table 3:** Correlation between age, scores of psychological distress, stigma, and attitude components (openness and value).

-	-	Age	Distress	Stigma	Openness
Distress	r_s_	-.102	-	-	-
	p	.001	-	-	-
	N	1076	-	-	-
Stigma	r_s_	-.034	.172	-	-
	p	.266	.000	-	-
	N	1077	1076	-	-
Openness	r_s_	.001	.063	-.100	-
	p	.966	.043	.001	-
	N	1047	1046	1047	-
Value	r_s_	.088	-.076	-.331	.273
	p	.004	.013	.000	.000
	N	1047	1046	1047	1047

**Table 4:** Factors related to distress, stigma, and attitude components.

		**Distress**						**Stigma**						**Openness**						**Value**					
		Mean	sd	Min	Max	MD	Mean	sd	Min	Max	MD	Mean	sd	Min	Max	MD	Mean	sd	Min	Max	MD
**Residence**	Aljouf	11.6	5.2	3	21	12	6.6	2.9	1	12	7	8.6	3.1	4	15	8	7.0	2.5	4	13	7
	Aseer	10.8	5.4	2	24	11	6.4	3.0	0	11	7	8.2	2.7	3	14	9	7.4	2.5	2	13	7
	Baha	10.5	3.3	6	16	10	6.1	4.1	0	13	7	8.3	3.2	4	14	8	7.8	2.2	5	12	8
	Eastern region	10.9	5.4	0	24	11	6.5	3.1	0	15	7	8.0	3.1	0	15	8	7.3	2.5	0	15	7
	Hail	11.9	1.9	9	14	13	8.4	2.4	4	11	9	7.7	2.5	5	11	7	6.9	1.5	4	8	7
	Jazan	10.0	5.5	0	24	10	6.8	3.1	0	15	7	8.6	2.7	0	15	9	7.0	2.6	0	15	7
	Madinah	10.6	4.7	0	18	11	6.4	3.6	0	13	7	9.3	3.4	3	15	10	6.4	2.7	0	12	6
	Makkah	11.3	5.2	1	23	10	5.8	2.8	0	12	6	8.6	2.2	2	13	9	7.4	2.7	1	13	7
	Najran	13.6	3.5	8	17	14	6.0	2.1	3	8	6	7.5	2.9	4	11	8	5.5	1.9	3	7	6
	Northern Border	10.1	6.7	0	24	11	5.3	4.7	0	15	5	7.4	3.1	3	11	9	6.1	2.1	3	10	6
	Qasseem	11.9	4.7	3	24	12	7.5	3.4	2	15	8	9.3	2.7	4	15	10	6.7	2.6	0	12	7
	Riyadh	9.5	5.1	0	24	9	6.6	3.0	0	15	7	7.9	2.6	0	15	8	7.0	2.3	0	15	7
	Tabuk	9.5	6.1	1	24	9	8.3	4.0	0	14	9	7.2	2.7	3	13	7	5.2	2.7	0	10	5
	p-value	.040					.124					.018					.012				
**Gender**	Male	9.1	5.0	0	24	9	6.9	3.1	0	15	7	7.8	2.8	0	15	8	6.9	2.5	0	15	7
	Female	11.6	5.3	0	24	12	6.2	3.1	0	15	7	8.7	2.7	0	15	9	7.2	2.5	0	15	7
	p-value	.000					.000					.000					.181				
**Marital status:**	Single	10.2	5.3	0	24	10	6.6	3.1	0	15	7	8.2	2.8	0	15	8	7.1	2.5	0	15	7
	Married	12.0	6.6	2	24	12	6.8	3.1	0	12	8	9.4	3.0	3	15	10	6.8	1.7	3	10	7
	Divorced	11.0	8.3	2	21	11	7.5	2.4	4	9	9	6.5	6.4	0	15	6	9.5	4.1	5	13	10
	Widowed	14.5	.7	14	15	15	6.0	1.4	5	7	6	7.5	3.5	5	10	8	4.5	.7	4	5	5
	p-value	.314					.763					.148					.206				
**Family monthly income (in Saudi riyals)**	<10000	10.5	5.1	0	24	11	6.8	2.9	0	15	7	7.8	2.7	0	14	8	6.8	2.5	0	14	7
	10000-20000	10.3	5.4	0	24	10	6.6	3.2	0	15	7	8.3	2.8	0	15	8	6.9	2.6	0	15	7
	> 20000	10.1	5.4	0	24	10	6.5	3.2	0	15	7	8.3	2.9	0	15	9	7.3	2.4	1	15	7
	p-value	.262					.341					.031					.018				
**Type of university**	Private	12.0	5.0	4	24	11	6.5	2.8	0	13	7	8.2	3.1	0	13	9	7.5	2.1	3	13	8
	Governmental	10.2	5.3	0	24	10	6.6	3.1	0	15	7	8.2	2.8	0	15	8	7.0	2.5	0	15	7
	p-value	.021					.686					.605					.125				
**Method of teaching**	PBL	10.2	5.3	0	24	10	6.6	3.1	0	15	7	8.1	2.8	0	15	8	7.1	2.5	0	15	7
	traditional	10.4	5.2	0	24	10	6.6	3.3	0	15	7	8.4	2.6	0	14	9	6.7	2.5	0	15	7
	p-value	.607					.577					.074					.010				
**Current year of study**	2ndyear	11.0	5.1	1	24	11	6.5	3.2	0	15	7	8.1	2.7	0	14	8	6.8	2.5	1	14	7
	3rd year	10.9	4.9	0	24	11	6.7	2.9	0	14	7	8.2	2.7	0	15	8	6.8	2.3	0	14	7
	4th year	11.0	5.8	0	24	11	6.9	3.3	0	15	7	8.5	2.9	0	15	9	6.9	2.8	0	15	7
	5th year	9.5	5.4	0	24	9	6.7	3.2	0	15	7	7.9	2.9	0	14	8	7.2	2.6	0	14	7
	6th year	9.5	5.4	0	24	9	6.3	3.0	0	15	7	8.2	2.7	0	15	8	7.3	2.4	0	15	7
	Internship	9.8	4.4	2	22	9	6.6	2.9	0	13	7	9.0	2.8	1	14	10	7.5	2.4	2	14	8
	p-value	.002					.654					.069					.066				
**Ever failed during med school**	Yes	12.1	5.1	0	24	12	7.1	3.1	0	15	7	8.1	2.7	1	15	8	7.0	2.7	0	14	7
	No	10.0	5.3	0	24	10	6.5	3.1	0	15	7	8.2	2.8	0	15	8	7.1	2.5	0	15	7
	p-value	.000					.038					.412					.959				

## Data Availability

The dataset generated and analyzed in this study is not publicly available due to its confidentiality nature. However, access may be granted by the Principal Investigator upon reasonable request, pending institutional ethical approval.

## LIST OF ABBREVIATIONS

GPA: Grade Point Average
K6: Kessler Psychological Distress Scale
PBL: Problem-Based Learning
PCA: Principal Component Analysis
SSRPH: A Stigma Scale for Receiving Psychological Help
ATSPPH-SF-A: Attitudes Toward Seeking Psychological Help

## STANDARDS OF REPORTING

STROBE guidelines were followed.
